# Cryo‐X‐Ray Phase Contrast Imaging Enables Combined 3D Structural Quantification and Nucleic Acid Analysis of Myocardial Biopsies

**DOI:** 10.1002/advs.202409163

**Published:** 2024-10-30

**Authors:** Kan Yan Chloe Li, Petros Syrris, Anne Bonnin, Thomas A Treibel, Vishwanie Budhram‐Mahadeo, Hector Dejea, Andrew C Cook

**Affiliations:** ^1^ Institute of Cardiovascular Science University College London London WC1N 1DZ UK; ^2^ Paul Scherrer Institut Villigen 5232 Switzerland; ^3^ Department of Cardiology St Bartholomew's Hospital London EC1A 7BE UK; ^4^ European Synchrotron Radiation Facility 71 Av des Martyrs Grenoble 3800 France

**Keywords:** biopsies, cryo‐X‐ray phase contrast imaging, genomics, nucleic acid analysis, transcriptomics

## Abstract

Snap‐frozen biopsies serve as a valuable clinical resource of archival material for disease research, as they enable a comprehensive array of downstream analyses to be performed, including extraction and sequencing of nucleic acids. Obtaining three‐dimensional (3D) structural information before multi‐omics is more challenging but can potentially allow for better characterization of tissues and targeting of clinically relevant cells. Conventional histological techniques are limited in this regard due to their destructive nature and the reconstruction artifacts produced by sectioning, dehydration, and chemical processing. These limitations are particularly notable in soft tissues such as the heart. In this study, the feasibility of using synchrotron‐based cryo‐X‐ray phase contrast imaging (cryo‐X‐PCI) of snap‐frozen myocardial biopsies is assessed and 3D structure tensor analysis of aggregated myocytes, followed by nucleic acid (DNA and RNA) extraction and analysis. It is shown that optimal sample preparation is the key driver for successful structural and nucleic acid preservation which is unaffected by the process of cryo‐X‐PCI. It is proposed that cryo‐X‐PCI has clinical value for 3D tissue analysis of cardiac and potentially non‐cardiac soft tissue biopsies before nucleic acid investigation.

## Graphical abstract

This study assesses the feasibility of using synchrotron‐based cryo‐X‐PCI for 3D structural analysis and nucleic acid extraction from snap‐frozen myocardial biopsies. It demonstrates that optimal sample preparation is critical for successful structural and nucleic acid preservation, suggesting clinical value for 3D tissue analysis before nucleic acid investigation.

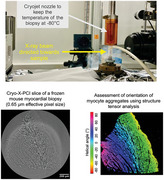

## 1 Introduction

Biopsies are essential for diagnosing and determining the extent of disease in clinical practice and research.^[^ ^1^ ^]^ To preserve tissue structure and prevent degradation, biopsies are typically placed in a fixative, such as formalin or glutaraldehyde. However, fixation can negatively impact nucleic acids through crosslinking of proteins, which in turn, can negatively affect the quality and accuracy of downstream analyses such as polymerase chain reaction (PCR) and single‐cell sequencing.^[^ ^2^, ^3^, ^4^, ^5^, ^6^, ^7^ ^]^ As a result, biopsies, including myocardial biopsies, are commonly taken during clinical research and diagnosis and snap frozen, preserving the molecular and cellular integrity of biopsies’ cells in their near “native state”, and allowing full range downstream ‐omics analyses to be performed, including those involving nucleic acids.

Conventional histology is considered the gold standard method for assessing the quality of ex‐vivo tissue at a microscopic level. While valuable, it is destructive and can distort the true 3D structure of tissues.^[^ ^8^ ^]^ There have been dramatic advancements in 3D structural analyses using techniques such as light sheet fluorescence microscopy (LSFM) which offer higher resolution and faster acquisitions.^[^ ^9^ ^]^ However, LSFM requires extensive sample preparation and optical clearing.^[^ ^10^ ^]^

Advanced techniques such as micro‐computed tomography (micro‐CT) and synchrotron‐based X‐ray phase contrast imaging (X‐PCI) can provide non‐destructive, 3D virtual histological information at high resolution.^[^ ^11^, ^12^, ^13^, ^14^, ^15^ ^]^ While micro‐CT can achieve a resolution as low as 10 µm, X‐PCI can potentially achieve below 1 µm.^[^ ^16^, ^17^ ^]^ These imaging methods are particularly useful for analyzing formalin‐fixed (FFPE) samples,^[^ ^11^, ^12^, ^15^, ^18^, ^19^, ^20^, ^21^, ^22^, ^23^, ^24^, ^25^, ^26^ ^]^ which are commonly used worldwide in clinical settings and biobanks as they are convenient for long‐term storage and preserve tissue at room temperature, and allow for histology and immunohistochemical staining.^[^ ^27^, ^28^, ^29^, ^30^ ^]^

Traditional, laboratory‐based micro‐CT has limited resolution and low contrast in soft tissues, such as the heart, and typically requires contrast agents. On the other hand, X‐PCI offers high‐resolution imaging of soft, low‐absorption‐contrast biological samples by detecting the phase shift as X‐rays pass through matter because of the differences in refractive index and tissue density. Previous studies have shown the effectiveness of X‐PCI for imaging formalin‐fixed and/or paraffin‐embedded myocardial biopsies from ex vivo human and animal hearts without sectioning nor staining while providing high‐resolution 2D and 3D virtual histopathology,^[^ ^14^, ^24^, ^31^ ^]^ thus enabling both morphological assessment^[^ ^14^, ^25^, ^26^, ^32^, ^33^ ^]^ and the visualization of sub(cellular) features.^[^ ^26^, ^34^ ^]^ Nucleic acid analysis is feasible for FFPE X‐PCI, however, formaldehyde fixation limits the range of techniques available and can increase the rate of errors during reverse transcription, leading to incorrect sequencing results.^[^ ^35^, ^36^, ^37^ ^]^ Therefore, a technique that enables 3D microstructural assessment of myocardium in its near‐native state whilst maintaining DNA and RNA integrity for subsequent downstream genomics and transcriptomics applications is highly desirable.

Cryo‐X‐PCI has been recently used to image frozen human meniscus tissue, where collagen fiber orientation could be assessed by structure tensor analysis, but nucleic acid analysis was not reported.^[^ ^38^ ^]^ Cryogenic contrast‐enhanced micro‐CT has been used to non‐destructively image skeletal muscle and tendon fibers in 3D but requires prior staining which can cause tissue shrinkage.^[^ ^39^ ^]^ Nucleic acid analysis was also not reported in their study. In this study, we propose that synchrotron‐based cryo‐X‐PCI can overcome these limitations allowing imaging of frozen myocardial biopsies for 3D morphologic analysis while preserving DNA and RNA integrity for downstream genomics and transcriptomics analyses.

## 2 Results

### 2.1 Cryo‐X‐PCI Enables 3D Morphological Assessment of Myocardial Biopsies in a Non‐Destructive Manner

**Figure 1:**
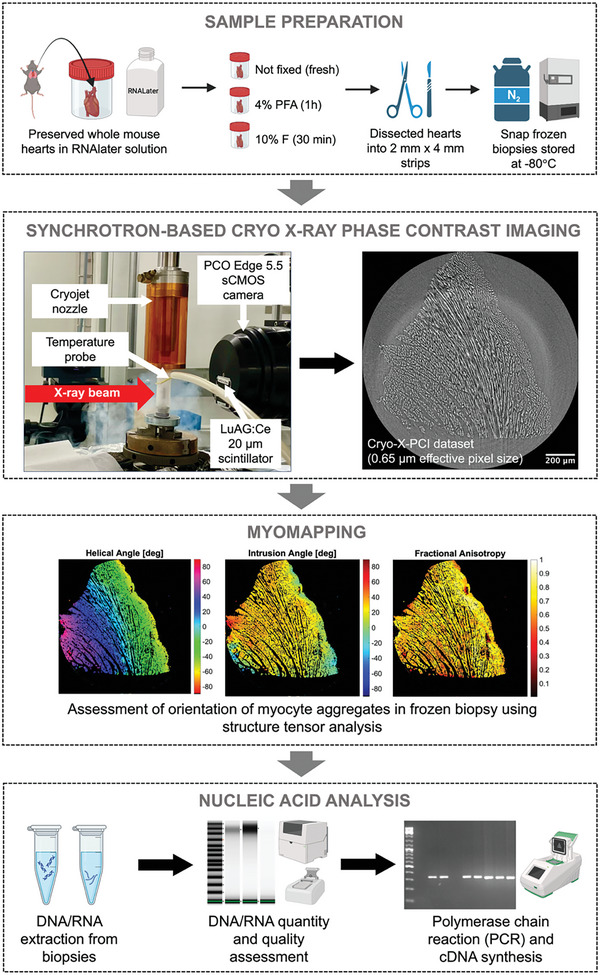
Schematic of synchrotron‐based cryo‐X‐PCI setup and workflow for combined 3D, non‐destructive myocardial morphologic assessment, and nucleic acid analysis.

**Figure 2:**
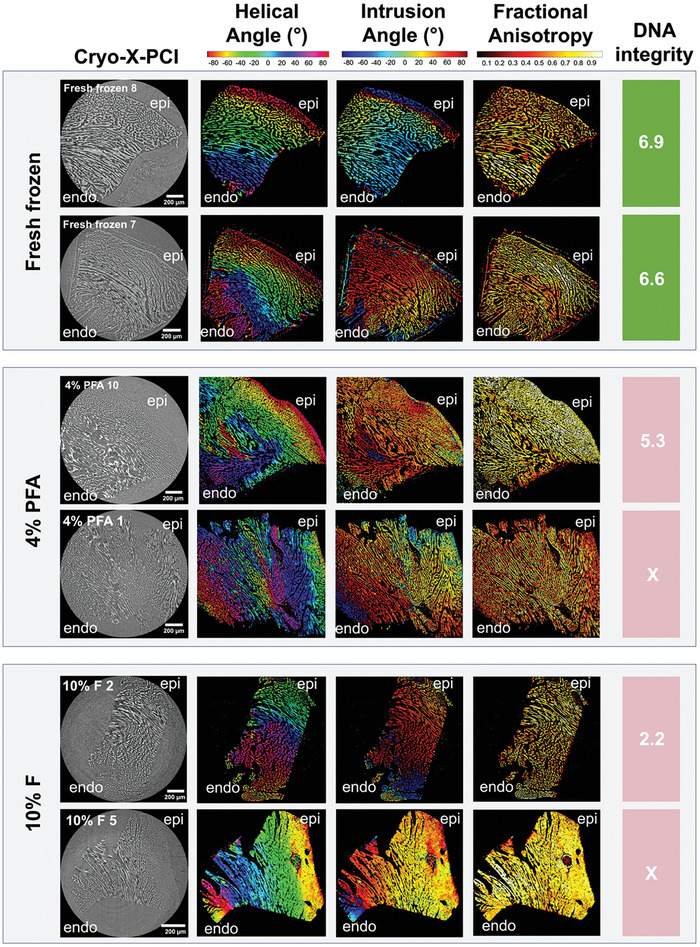
Myomapping and DNA integrity in representative fresh frozen, 4% paraformaldehyde‐fixed (4% PFA) and 10% formaldehyde‐fixed (10% F) mouse myocardial biopsies after cryo‐X‐PCI. An expected gradual change in HA could be observed, from positive HA in the endocardium (endo) to negative HA in the epicardium (epi).

30 myocardial mouse samples were investigated using a dedicated cryo‐X‐PCI setup at the TOMCAT beamline (**Figures** **1**, and S1, Supporting Information). Using the structure tensor (ST) method, gradual changes in helical angle (HA) and intrusion angle (IA) from endocardium (endo) to epicardium (epi) could be visualized and quantified in 16 samples (**Figure** **2**, Figures S4–S6, Supporting Information). The myocyte aggregates in these biopsies were aligned in a way that showed a gradual change in HA from positive angulation in the endocardium to negative angulation in the epicardium (Figure 2, Figures S4–S6, Supporting Information). Spacing between myocytes was increased in the majority of samples.

Of the remainder (5 fresh frozen, seven 4% PFA, and two 10% F biopsies), myomapping was sub‐optimal due to artifacts from large ice crystals. Ice crystal artifacts were also observed in control mouse myocardial biopsies (Figure S7, Supporting Information). Overall myocardial morphology was better preserved in 10% F samples, than fresh frozen biopsies (Figures S4 and S5, Supporting Information). 4% PFA samples showed unusual disruption to morphology, suggestive of an interaction between RNA later and PFA, along with artifacts due to ice crystal formation (Figure S6, Supporting Information).

Cryo‐sectioning and H&E‐staining of 8 cryo‐X‐PCI samples plus 2 control samples were feasible after cryo‐X‐PCI without tissue damage (Figure S8, Supporting Information). Fractures to myocytes were observed in regions of ice crystal formation in both cryo‐X‐PCI and controls (Figure S8, Supporting Information) along with variation in spacing between myocytes in preserved regions.

### 2.2 Cryo‐X‐PCI Does Not Affect DNA or RNA Integrity

**Figure 3:**
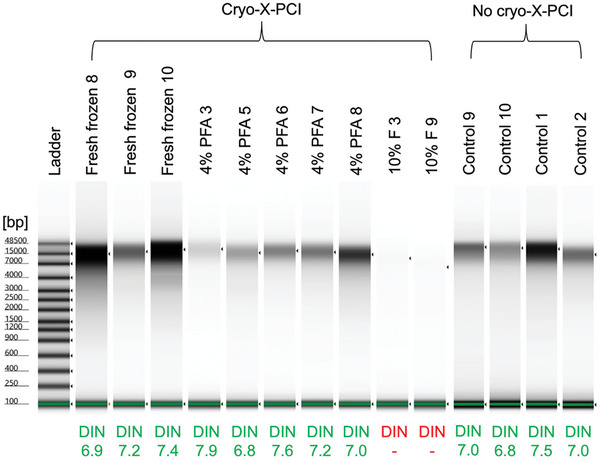
DNA quality control TapeStation gels with DNA Integrity Number (DIN) values of gDNA extracted from representative mouse myocardial biopsies after synchrotron‐based cryo‐X‐PCI. Key: 4% PFA = 4% PFA‐fixed samples; 10% F = 10% formaldehyde‐fixed samples. Control samples represent those that were collected in RNAlater solution then snap frozen in liquid nitrogen and did not have cryo‐X‐PCI. DIN values appropriate for downstream analysis are shown in green. DIN values unsuitable for downstream analysis are shown as red including those that could not be calculated due to very low concentrations of DNA.

Both DNA and RNA were extracted from all samples (22 mouse myocardial samples with cryo‐X‐PCI and 8 control samples without cryo‐X‐PCI) (Table S3, Supporting Information). DNA integrity was highest for fresh frozen samples (without fixation), with mean DNA Integrity Number (DIN) values of 7.0 (with cryo‐X‐PCI) and 7.2 (control) and a range of 0.9 (cryo‐X‐PCI) and 1.0 (control) (Table S3, Supporting Information). All fresh frozen samples had DIN values that exceeded the minimum cut‐off (DIN > 6, **Figure** **3** and Table S3, Supporting Information).^[^ ^49^ ^]^ In 4% PFA‐fixed samples, mean DIN values were lower (mean 6.9, range 2.6), and were the lowest for 10% F samples (mean 3.0, range 1.6) (Figure 3 and Table S3, Supporting Information).

Further assessment of DNA integrity was achieved through sequence‐specific polymerase chain reaction (PCR) amplification of exon 28 of mouse myosin binding protein C3 (Mybpc3). All fresh frozen and 4% PFA‐fixed DNA samples were of sufficient quality and integrity to amplify the exon successfully with visualization of the expected amplicon PCR product (330 base pairs, bp) (Figure S9, Supporting Information). In contrast, 10% F samples showed weak PCR product bands (Figure S9, Supporting Information).

RNA Integrity Number (RIN) values were significantly lower than corresponding DIN values. Fresh frozen samples had the highest RIN values overall (mean RIN value of fresh frozen sample = 4.4; range of 5.0), followed by 4% PFA samples (mean = 3.8; range of 3.1), and 10% F samples (mean = 2.9; range of 1.6) (Figure S10, Table S3, Supporting Information). The control samples (those that did not undergo cryo‐X‐PCI) also had low RIN values (mean RIN value of 4.0; range of 4.4) where only one sample exceeded a RIN value of 6 (sample: control 9) (Figure S10, Table S3, Supporting Information). Further assessment of RNA integrity was performed through reverse transcription PCR of extracted RNA (Figure S11, Supporting Information). Despite the low RNA concentrations and RIN values, the extracted samples could still be successfully reverse‐transcribed to cDNA and subsequently amplified by PCR (Figure S11, Supporting Information).

## 3 Discussion

Snap‐frozen biopsies represent a vast resource for clinical research as they have superior molecular integrity for performing the full array of downstream nucleic acid analysis compared to formalin‐fixed tissue. However, obtaining 3D structural information from frozen tissue before analysis is challenging. In contrast, formalin‐fixed tissue is the standard for obtaining 2D structural information. Formalin‐fixed tissue is less compatible with nucleic acid extraction and analysis, and tissue distortion through histologic processing still occurs, which is particularly relevant to 3D analysis of complex, soft tissues, such as myocyte arrangement in the myocardium. A procedure that can combine imaging of (snap‐frozen) tissue, in its near‐native state, and be followed by nucleic acid analysis could therefore be clinically relevant for combined in‐depth characterization and targeting of disease.

In this study, we demonstrate the feasibility of synchrotron‐based cryo‐X‐PCI of frozen myocardial biopsies prepared under differing conditions. We describe, for the first time, the combination of 3D structural analysis from synchrotron‐based cryo‐X‐PCI with assessment of nucleic acid analysis, focussing particularly on DNA and RNA integrity post‐cryo‐X‐PCI.

As a marker of high‐level morphologic preservation, we demonstrate that quantification of myocyte orientation (myomapping) in frozen myocardial biopsies is feasible through structure tensor analysis and that HA, IA, and FA morphologic parameters could be assessed. Myomapping showed a gradual change in HA, from positive HA in the endocardium to negative HA in the epicardium, and is consistent with previously reported studies that have quantified myocyte orientation in whole hearts, in both animal models and human samples.^[^ ^45^, ^46^, ^50^ ^]^ With biopsies, there is an additional layer of complexity in that the orientation of the biopsy is not always known. To mitigate this, datasets were carefully oriented with respect to the epicardium and endocardium before performing myomapping. We also compared cryo‐X‐PCI with 2D traditional histology through cryo‐sectioning and H&E staining and confirmed similar appearances before and after imaging. The increased spacing between myocyte aggregates found in our frozen biopsies closely resembles that reported in frozen tendon tissue imaged by cryogenic contrast‐enhanced micro‐CT^[^ ^39^ ^]^ suggesting that this feature is inherent to imaging tissues in their frozen state. Ice crystal artifacts were present in some biopsies, including in the control samples which is not ideal. Despite the presence of freezing artifacts, quantification of the orientation of myocyte aggregates was still feasible in cryo‐X‐PCI samples as indicated by the myomapping results. Our study was designed to replicate the freezing process that is commonly used in clinical practice which typically involves the rapid collection of biopsies in a cryogenic tube and snap‐freezing them in liquid nitrogen. However, other techniques for snap‐freezing samples could be investigated in the future.

Importantly, our study also shows that DNA and RNA can be successfully extracted from cardiac biopsies following cryo‐X‐PCI. Fresh frozen samples had optimal recovery of DNA as indicated by the DIN values greater than 6 for all extracted DNA samples followed by lower DIN values for biopsies preserved in 4% PFA and 10% formalin. Sequence‐specific (Mybpc3 exon 28) PCR was successful for all fresh frozen and 4% PFA‐fixed samples which all showed the 330 bp PCR product band. The 10% F samples had the lowest integrity as indicated by the low DIN values and only one faint Mybpc3 exon 28 PCR product band could be observed. Most likely this was due to extensive crosslinking from 10% formaldehyde fixation and agrees with our preliminary study.^[^ ^25^ ^]^

Low RIN values from TapeStation and Nanodrop measurements for the extracted RNA from biopsies were observed. Nonetheless, we optimized RT‐PCR conditions to account for the small biopsy size and low RNA concentrations and showed that RNA could still be reverse‐transcribed to cDNA. The cDNA concentrations were very low and had to be re‐amplified for the bands to show up clearly on the gel, but they were still viable for gene‐specific PCR amplification as shown by the success of Mybpc3 exon 28 PCR.

We also compared DIN and RIN values for cryo‐X‐PCI biopsies with control biopsies (those that did not undergo cryo‐X‐PCI). There were no significant differences between the mean DIN and RIN values for fresh frozen samples that had cryo‐X‐PCI (mean DIN = 7.0, mean RIN = 4.4) compared to the fresh frozen samples that did not undergo cryo‐X‐PCI (mean DIN = 7.2, mean RIN = 4.0). We found no significant differences in nucleic acid quantity and quality, suggesting again that the small size of the biopsy was the limiting factor instead of cryo‐X‐PCI itself. Therefore, overall, if the goal is to have high nucleic acid integrity for downstream genomics and transcriptomics applications, such as sequencing, it would be best to avoid fixing samples, and simply prepare biopsies with optimal preservation of DNA and RNA, followed by freezing. On the other hand, if the aim is to have superior myocardial morphological detail but not perform subsequent omics, then fixing the biopsies in formaldehyde and processing them into FFPE blocks would be sufficient.

To conclude, cryo‐X‐PCI can provide a non‐destructive 3D assessment of myocardial morphology and can be combined with nucleic acid analysis of frozen biopsies. Synchrotron‐based cryo‐X‐PCI does not appear to affect DNA or RNA integrity for downstream genomics and transcriptomics applications, and we recommend fresh frozen sample preparation for optimal results in both morphology and nucleic acid quality. Although we only tested cryo‐X‐PCI in mouse myocardial biopsies, this technique has the potential to be integrated into the clinical setting as a technique to image frozen biopsies from a range of diseases. We foresee the clinical potential of cryo‐X‐PCI to examine further resources of snap frozen material (both cardiac and non‐cardiac soft tissue) to provide 3D virtual histopathology and correlation between structural information and genetics.

Our study demonstrates the potential of cryo‐X‐PCI for non‐destructive, 3D structural analysis of myocardial biopsies, followed by nucleic acid extraction and analysis. However, several limitations were encountered. The snap freezing process, designed to replicate clinical practice, was not optimized to minimize the use of animal tissues and beamtime. Future studies should explore methods to reduce freezing artifacts, such as employing liquid nitrogen‐cooled isopentane or hexane, which are known to provide more controlled freezing conditions without causing significant freezing artifacts. Notably, this can further complicate sample collection in surgical settings, but their use could significantly improve the quality of frozen samples.

While our study focused on DNA and RNA integrity as markers of nucleic acid analysis preservation, protein integrity was not assessed. However, we have previously shown that X‐PCI does not inherently affect protein epitopes as seen via immunohistochemistry. The availability of synchrotron beamtime, which is both competitive and expensive, limited our study to mouse myocardial biopsies, which were used to demonstrate proof‐of‐concept before valuable human myocardial biopsies could be used. This meant that a specific disease state or pathology was not studied in this paper but should there be cryo‐X‐PCI beamtime in the future, then future work would apply this technique for imaging frozen myocardial biopsies from heart muscle disease or cardiomyopathy patients to further prove the clinical relevance of this technique.

Currently, cryo‐X‐PCI is a research technique confined to specialized synchrotron facilities. However, recent advancements in tabletop X‐ray phase contrast imaging may offer promising alternatives in the future. These compact systems have demonstrated the ability to perform high‐resolution phase contrast imaging without the need for synchrotron facilities.^[^ ^51^, ^52^ ^]^ Nonetheless, benchtop facilities have limitations in terms of their resolution and the size of samples they can accommodate. Developing tabletop cryo‐X‐PCI could significantly expand clinical relevance. By integrating cryo‐sources with benchtop phase contrast imaging, detailed 3D structural information could be obtained from myocardial biopsies of patients with heart muscle disease or cardiomyopathy. This would provide valuable insights into disease pathology and progression, and could also be applied to non‐cardiac biopsies. The development of benchtop phase contrast instruments would facilitate the integration of cryo‐X‐PCI into clinical practice, allowing for the routine analysis of biopsies in a more efficient, cost‐effective, and accessible manner. Ultimately, this could result in improved diagnostic capabilities and personalized treatment strategies for patients. To further validate the clinical feasibility and utility of cryo‐X‐PCI, future studies should focus on imaging frozen myocardial biopsies from patients with specific disease states or pathologies. By doing so, cryo‐X‐PCI could be established as a valuable tool for clinical research and diagnostics, ultimately improving patient outcomes.

## 4 Experimental Section

### Sample Description and Preparation

All animal studies were approved by the University College London Biological Services Ethical Review Committee and performed with UK Home Office approval (Project Licence number PB12FFA7E). Animal work conformed to the UK Animals (Scientific Procedures) Act 1986. All tissue samples collected from this study were from excess wild‐type C57BL/6 mice. Four wild‐type C57BL/6 male mice were sacrificed using the approved Schedule 1 humane killing method by anesthetizing them via carbon dioxide gas overdose in a rising concentration at 20% flow rate for 5 min followed by an additional 2 min to ensure death. For confirmation of death, dislocation of the neck was performed. Their hearts were rapidly removed and collected in RNAlater^TM^ Stabilisation Solution (Thermo Fisher Scientific) at room temperature. Two of these hearts were snap‐frozen using liquid nitrogen (fresh frozen). One heart was fixed in 4% paraformaldehyde (4% PFA) for 1 h. The remaining heart was fixed in 10% formaldehyde (10% F) for 30 min. After fixation, 10 small biopsies of myocardium were dissected from each heart to give a total of 40 myocardial biopsies. The myocardial biopsies were ≈2 mm x 4 mm in dimensions.

Each myocardial biopsy was carefully drawn along with OCT mounting media (VWR Chemicals) into a 1 mL syringe to avoid bubbles. The end of the syringe was positioned on the central spindle of a Magnetic CryoCap^TM^ (Molecular Dimensions MD7‐400) (Figure S1a, Supporting Information) before snap freezing with liquid nitrogen. Myocardial biopsies were protected by Magnetic CryoVials^TM^ (Molecular Dimensions MD7‐402) and stored at −80 °C until required.

### Synchrotron‐Based Cryo‐X‐Ray Phase‐Contrast Imaging (cryo‐X‐PCI)

Thirty samples (n = 10 fresh frozen, n = 10 4% PFA, n = 10 10% F) were transported on dry ice to the Paul Scherrer Institute (Villigen, Switzerland), where synchrotron‐based cryo‐X‐PCI was performed at the TOMCAT X02DA beamline of the Swiss Light Source using an in‐house developed setup (Figure S1b, Supporting Information). The remaining 10 fresh frozen myocardial biopsies used in this study did not undergo cryo‐X‐PCI and served as controls for comparison between cryo‐X‐PCI versus traditional 2D histology (cryo‐sectioning).

Each sample was carefully positioned on a dedicated magnetic mount on the rotation stage to allow for fast sample positioning and exchange. The sample was placed under a double‐walled orange Kapton foil cage mounted on a cryo jet nozzle and kept at −80 °C. The flow of cold nitrogen gas was obtained from a Cryojet5 (Oxford‐Instruments). The cage was positioned directly above the CryoVial to keep the biopsy frozen throughout the scan and avoid freeze‐thaw effects (Figure S1b, Supporting Information). After centering the sample on the rotation stage, tomographic acquisition was performed using the standard X‐ray microscope setup. The microscope was composed of a LuAG:Ce scintillator screen of 20 µm, an x10 magnification objective, and a PCO.Edge 5.5 CMOS camera. The field of view was 1.7 × 1.4 mm^2^ and the effective pixel size was 0.65 µm. 1000 projections were captured over 180° using a 50 ms exposure time per projection, a beam energy of 21 keV, and a propagation distance of 60 mm (Table S1, Supporting Information).

### Image Reconstruction and Visualization

After the acquisition, all projections were corrected with dark and flat‐field images. Phase retrieval was performed with the Paganin phase retrieval filter algorithm (δ/β = 5.3e‐7/9.3e‐9 = 57) before reconstruction in 3D by using the Gridrec algorithm.^[^ ^40^, ^41^ ^]^ Each scanned volume of interest was saved as a volumetric dataset comprised of 2160 image slices at 16‐bit pixel depth. Visual inspection of myocardial morphology was performed using Fiji/ImageJ (ImageJ, version 1.51, Wayne Rasband).^[^ ^42^ ^]^ Datasets were reduced from 16‐bit to 8‐bit depth and cropped to fit the biopsy size accordingly to remove redundant data and reduce computational costs during image processing.

### Quantification of Myocyte Orientation (“myomapping”)

To quantify the orientation of myocyte aggregates (“myomapping”), structure tensor (ST) analysis was used where helical angle and intrusion angle were computed with an in‐house developed MATLAB script.^[^ ^11^, ^43^, ^44^, ^45^, ^46^ ^]^ The ST was calculated at each voxel using prolate spheroidal coordinates (*λ, μ, θ*), which provide more accurate representation of the 3D orientation of cardiomyocyte aggregates than Cartesian coordinates.^[^ ^46^ ^]^ Eigen‐decomposition of the ST at each voxel yielded its three eigenvalues and their eigenvectors, which represent the magnitude and direction of orientation of cells, respectively. The tertiary eigenvector, which has the smallest eigenvalue, represents the vector following the orientation of myocyte aggregates in their longitudinal axis due to correspondence with the lowest intensity variation (Figure S2, Supporting Information).^[^ ^44^, ^46^, ^47^ ^]^ HA represents the longitudinal direction of myocyte aggregates with respect to the long axis of the ventricle, while intrusion angle (also known as transverse angle) describes the angle at which the myocyte aggregates penetrate the myocardium on the cross‐sectional plane. Fractional anisotropy (FA) is the degree of anisotropy or disorganization of the local myocardium. To focus ST analysis on relevant areas and exclude background, a segmentation mask was created for each dataset using a semi‐automatic pixel classification workflow in the open‐source software Ilastik (Figure S3, Supporting Information).^[^ ^48^ ^]^

### Traditional 2D Histologic Assessment

Eight myocardial samples were used for cryo‐sectioning after cryo‐X‐PCI: four fresh frozen samples (fresh frozen 3, 4, 5, and 6), two 4% PFA‐fixed samples (4% PFA 4 and 9), and two 10% formalin‐fixed samples (10% F 4 and 8). Two fresh frozen samples that did not have cryo‐X‐PCI (controls 7 and 8) served as controls and underwent cryo‐sectioning. Cryo‐sections of 8 µm thickness were cut with a cryostat (Leica) at −20°C and collected on SuperFrost^TM^ Plus microscope slides (VWR). Haematoxylin and eosin (H&E) frozen staining protocol was used to stain cryo‐section slides via an automated system (Tissue‐Tek DRS 2000 Multiple Slide Stainer, Sakura) (Table S2, Supporting Information). Stained slides were digitized using a Nanozoomer Whole Slide Imager and viewed with NDP.View 2 software (Hamamatsu Photonics).

### Extraction, Quality Assessment, and Quantitation of DNA and RNA

After synchrotron‐based cryo‐X‐PCI, genomic DNA (gDNA) and total RNA were extracted from 30 mouse myocardial biopsies using AllPrep DNA/RNA/miRNA Universal Kit (catalog number 80224, Qiagen, Germany) following the standard manufacturer's protocol. The concentration and purity of DNA were quantified using NanoDrop™ Lite Spectrophotometer (Thermo Fisher Scientific, USA) and measured with NanoDrop spectrophotometer using A_260_/A_230_ and A_260_/A_280_ absorbance ratios. Quality control of extracted DNA and RNA was performed using Agilent TapeStation 2200 (UCL Genomics) to assess fragmentation and DNA integrity number (DIN) and RNA integrity number (RIN) values. DIN and RIN values range from 1 (highly degraded DNA/RNA) to 10 (highly intact DNA/RNA).^[^ ^49^ ^]^

The suitability of the extracted DNA to be used as template in PCR was assessed on exon 28 of the mouse myosin binding protein C3 (Mybpc3) gene using the following primer sequences: 5′ AGCTATAGTGCTCTGGACCCT 3′ (forward primer) and 5′ CCCAACCCTGAGCTTGACG 3′ (reverse primer) with AmpliTaq Gold^TM^ DNA polymerase (Applied Biosystems^TM^). Agilent Technologies SureCycler 8800 thermal cycler was used for PCR with the following conditions: initial hot start (96 °C, 10 min) followed by 35 cycles of denaturation (96 °C, 30 s), annealing (59 °C, 1 min) and elongation (72 °C, 1 min), and a final elongation step (72 °C, 7 min). PCR products were separated using agarose gel electrophoresis (2% w/v) and the expected band at 330 base pairs (bp) was detected by an UV light transilluminator (GelDoc imaging system) following staining with GelRed (#41 003, Biotium).

Extracted RNA (11 µL) from myocardial biopsies was reverse transcribed with the SuperScript^TM^ IV First‐Strand Synthesis System kit (Invitrogen) using Mybpc3 exon 28 reverse primer (5′ CCCAACCCTGAGCTTGACG 3′). RNA‐primer mix was heated at 65 °C (5 min) and then incubated on ice for at least 1 min. The reverse transcriptase reaction and RNA‐primer mix were incubated at 50 °C (20 min) and then inactivated at 80 °C (10 min). PCR on exon 28 of Mybpc3 gene was performed with the aforementioned mouse primer sequences using 8 µL cDNA in a total reaction volume of 25 µL per sample with the following conditions: initial hot start (96 °C, 10 min) followed by 40 cycles of denaturation (96 °C, 1 min), annealing (58 °C, 1 min) and elongation (72 °C, 2.5 min), and a final elongation step (72 °C, 7 min).

## Conflict of Interest

The authors declare no conflict of interest.

## Author Contributions

H.D. and A.C C. contributed equally to this work as senior authors. A.C.C., P.S., T.T., and K.Y.C.L. conceptualized the project. K.Y.C.L. performed DNA and RNA extraction from myocardial biopsies, cDNA synthesis, PCR, and RT‐PCR under the supervision and guidance of P.S. K.Y.C.L. performed myocyte orientation analysis under the supervision and guidance of H.D. A.B. assisted with cryo‐X‐PCI setup design, beamtimes, and tomographic reconstructions. K.Y.C.L. wrote the manuscript under the guidance and supervision of A.C.C., H.D., and P.S. All authors assisted in reviewing and revising the manuscript.

## Supporting information



Supporting Information

## Data Availability

The data that support the findings of this study are available from the corresponding author upon reasonable request.
