# Supplementary material for: Cryo‐X‐Ray Phase Contrast Imaging Enables Combined 3D Structural Quantification and Nucleic Acid Analysis of Myocardial Biopsies
Source: Adv Sci (Weinh). 2024 Oct 30;11(48):2409163. doi: 10.1002/advs.202409163 (PMC11672302; doi:10.1002/advs.202409163)
Supplement: Supplementary file 1 — Supporting Information [file ADVS-11-2409163-s001.pdf]

ADVANCED SCIENCE
Open Access

Supporting Information

for *Adv. Sci.*, DOI 10.1002/advs.202409163

Cryo-X-Ray Phase Contrast Imaging Enables Combined 3D Structural Quantification and Nucleic Acid Analysis of Myocardial Biopsies

Kan Yan Chloe Li, Petros Syrris, Anne Bonnin, Thomas A Treibel, Vishwanie Budhram-Mahadeo, Hector Dejea and Andrew C Cook*

Supplementary Information

Supplementary Figures

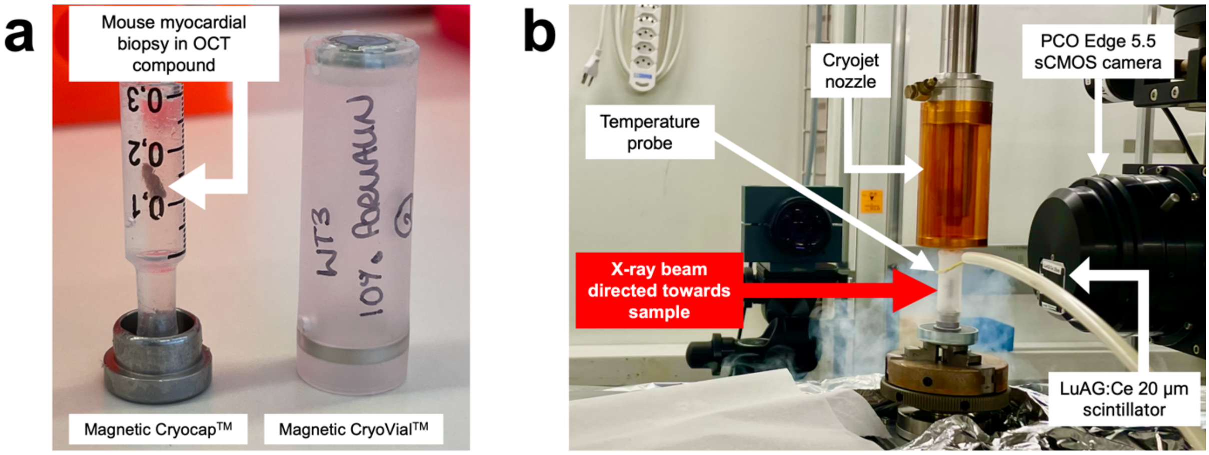

Figure S1 | Sample mounting and synchrotron-based cryo-X-PCI setup used to image frozen mouse myocardial biopsies. a, Myocardial biopsies were mounted in OCT mounting media (VWR Chemicals) within a 1 ml syringe placed on the central spindle of a Magnetic CryoCap™ (Molecular Dimensions MD7-400) and protected by Magnetic CryoVials™ (Molecular Dimensions MD7-402). **b,** Cryo-X-PCI setup at the TOMCAT X02DA beamline (Swiss Light Source), comprised by a cryojet nozzle positioned above the biopsy, a temperature probe, and a detection system (20 µm LuAG:Ce scintillator, 10x and PCO.Edge 5.5 MOS camera).

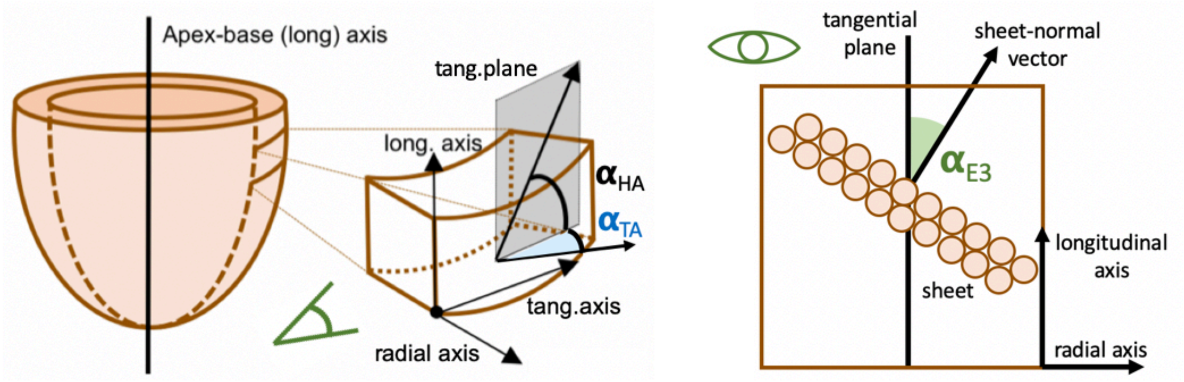

Figure S2 | Schematic of structure tensor and angles used for quantification of myocyte orientation ("myomapping"). Gradient-based structure tensor analysis was performed with an in-house MATLAB script as described in previous studies[44,46,47].

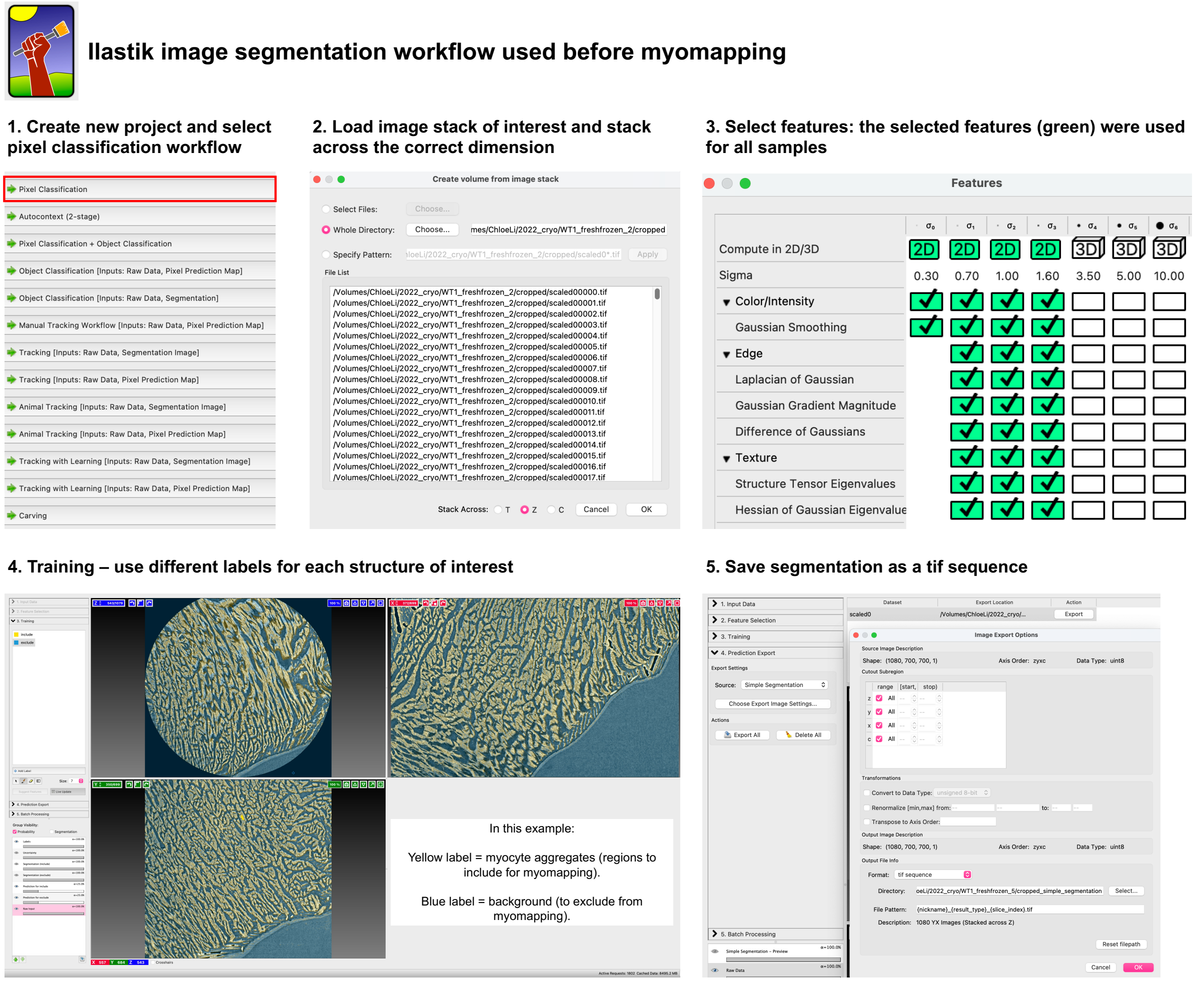

Figure S3 | Pixel classification workflow used for segmentation of cryo-X-PCI datasets in Ilastik[48] before myomapping was performed. A segmentation mask was created for each dataset to focus structure tensor analysis to relevant areas and exclude background.

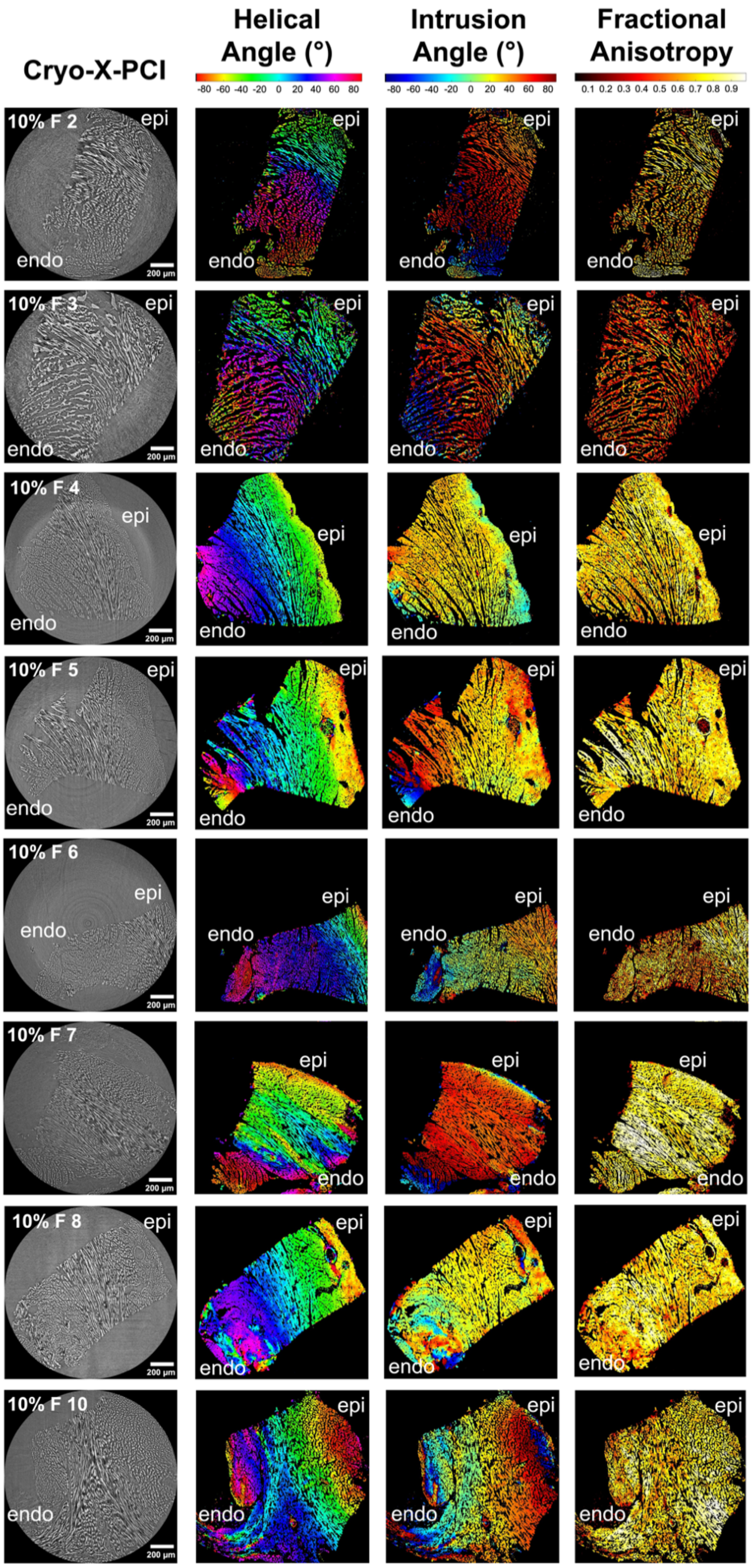

Figure S4 | Quantification of myocyte orientation (myomapping) in 10% formaldehyde-fixed (10% F) mouse myocardial biopsies.

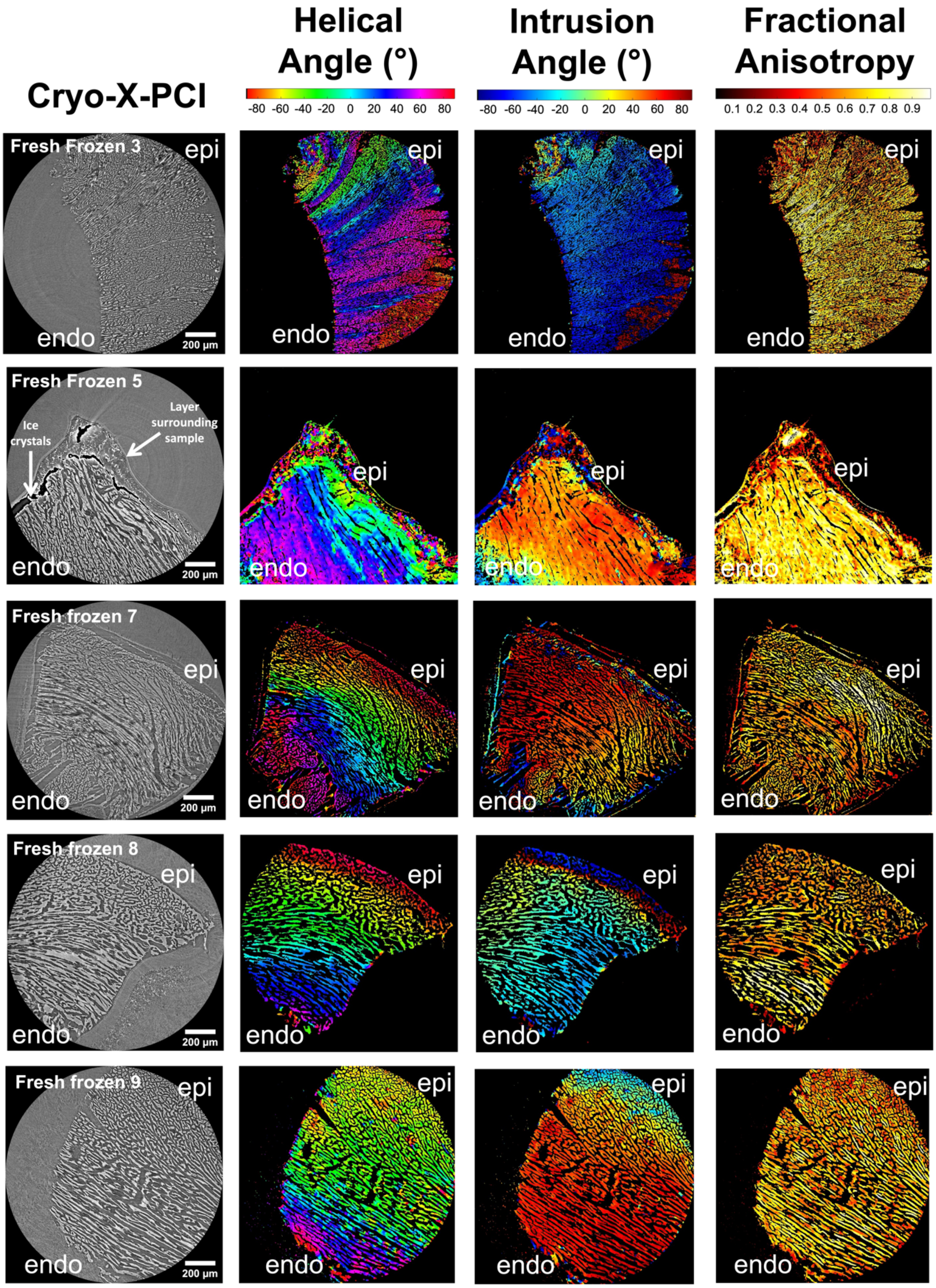

Figure S5 | Quantification of myocyte orientation (myomapping) in fresh frozen mouse myocardial biopsies.

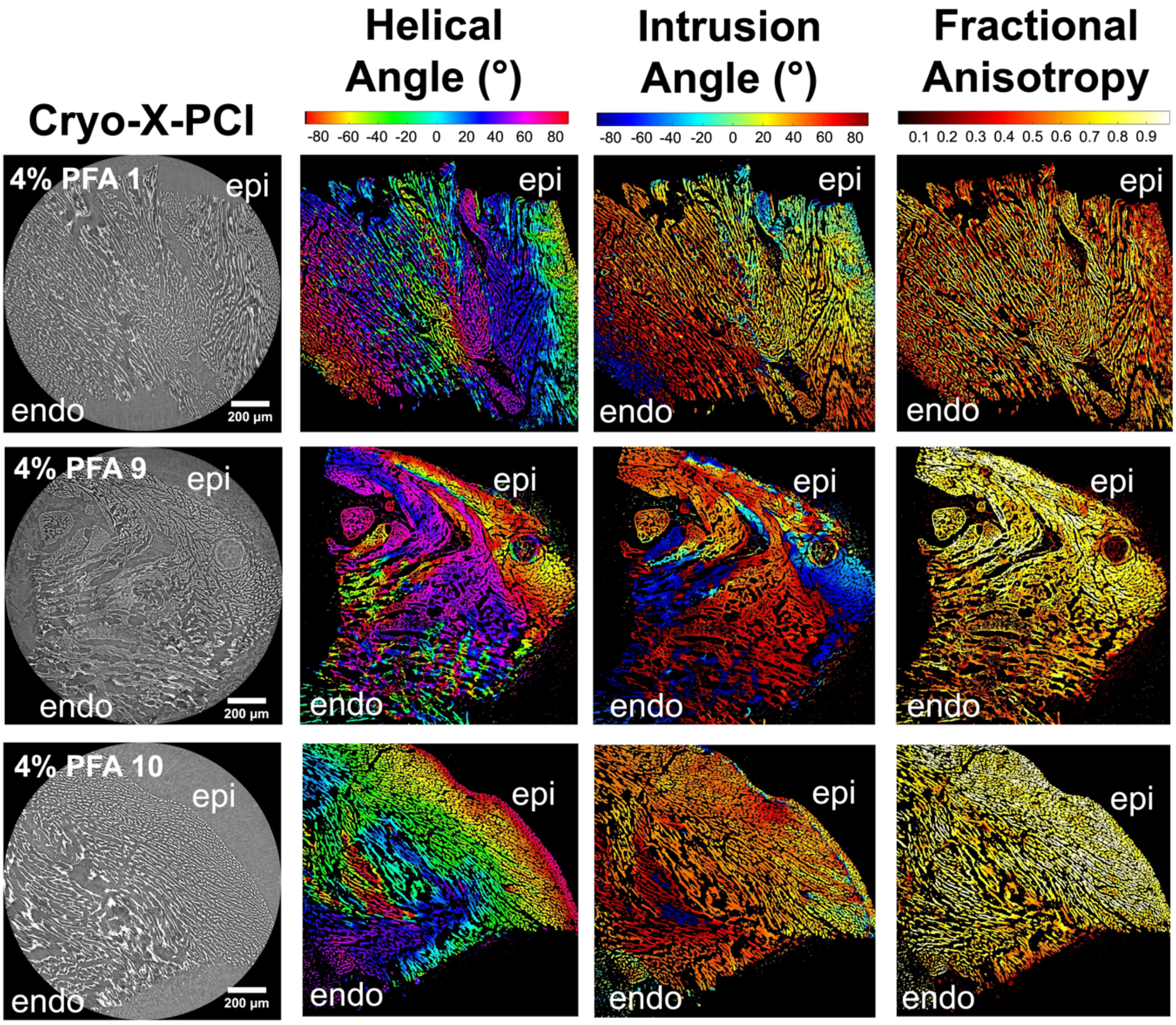

Figure S6 | Quantification of myocyte orientation (myomapping) in 4% paraformaldehyde (4% PFA) mouse myocardial biopsies.

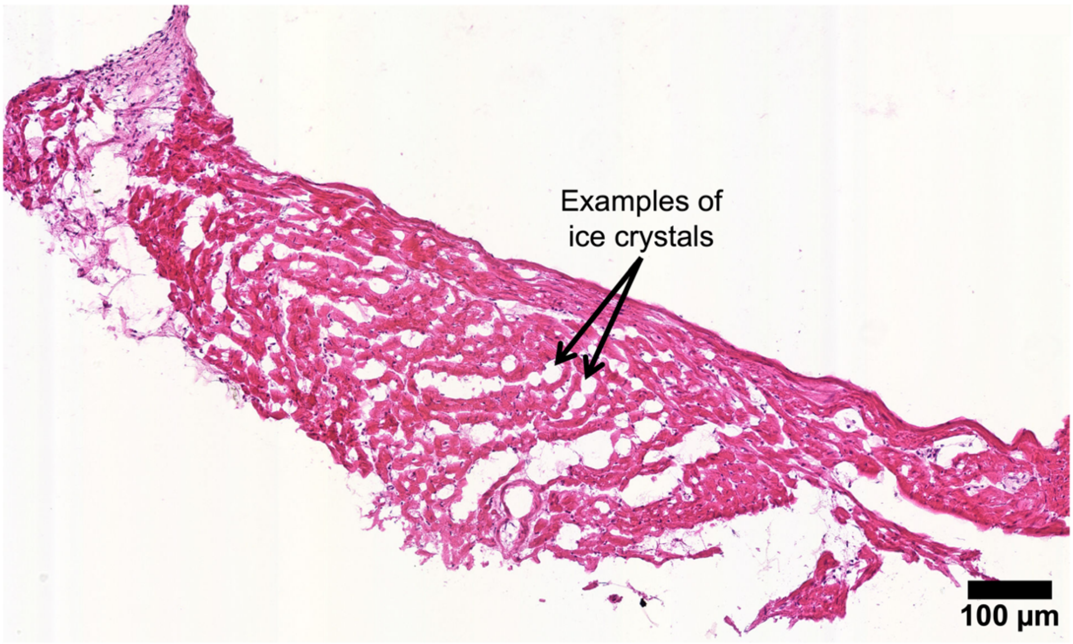

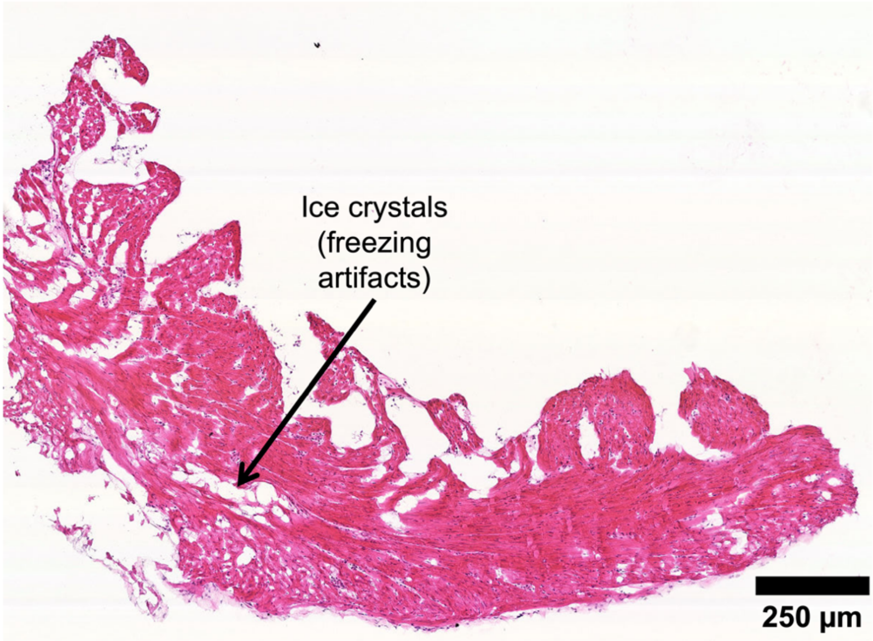

Figure S7 | Ice crystal artefacts in representative cryo-sections from control (no cryo-X-PCI) mouse myocardial biopsies. Cryo-sections of 8 µm thickness were cut at -20°C and collected on SuperFrost^{TM} Plus microscope slides (VWR). A haematoxylin and eosin (H&E) frozen staining protocol was used to stain cryo-section slides via an automated system (Tissue-Tek DRS 2000 Multiple Slide Stainer, Sakura) (**Table S2**).

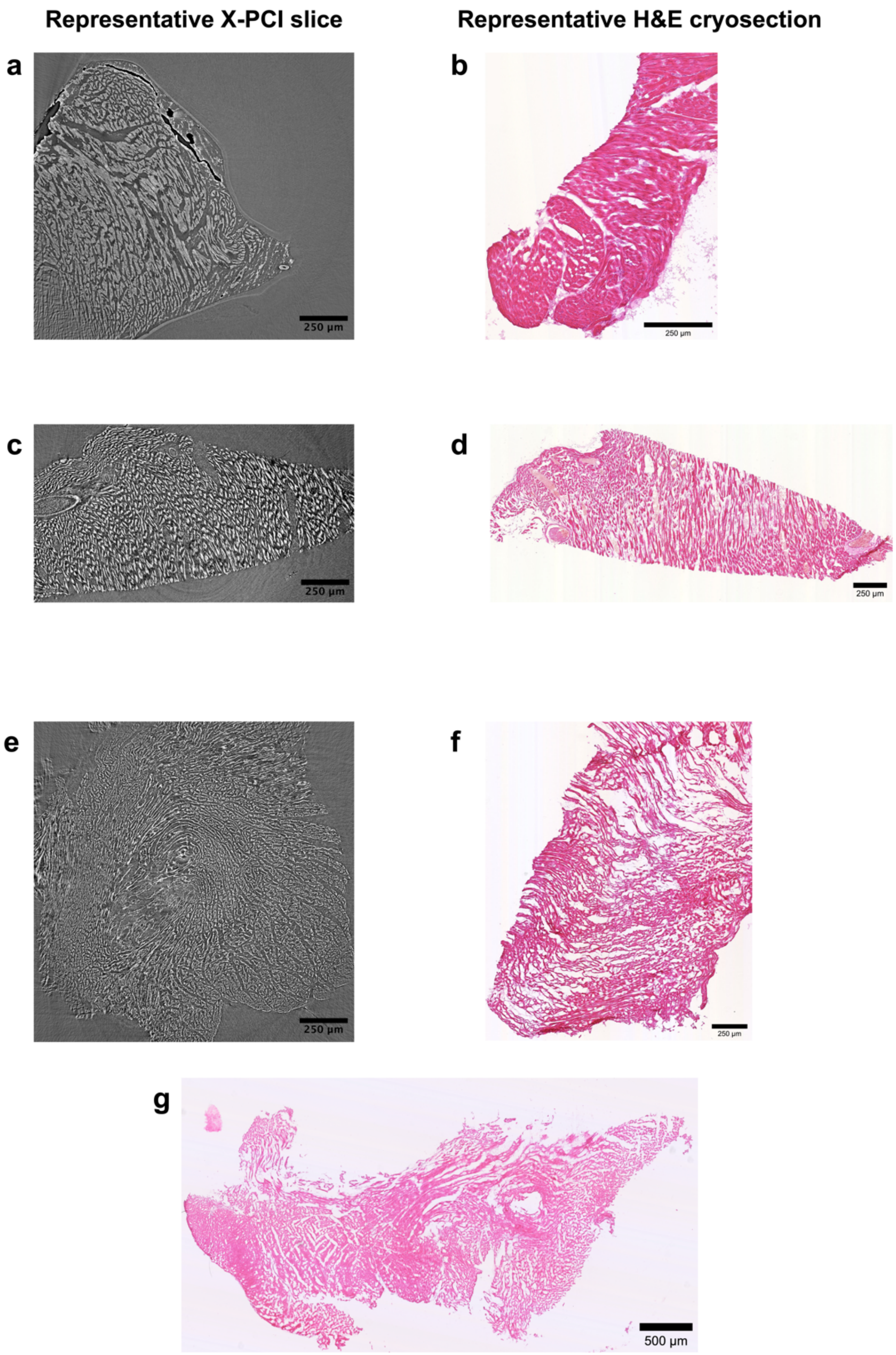

Figure S8 | Representative cryo-X-PCI images showing increased spacing between myocytes with corresponding H&E cryo-sections. a-b, fresh frozen, **c-d,** 10% formaldehyde-fixed, **e-f,** 4% PFA-fixed, **g,** control. The left column shows representative cryo-X-PCI slices. Right column shows representative haematoxylin and eosin (H&E)-stained sections from corresponding myocardial biopsies.

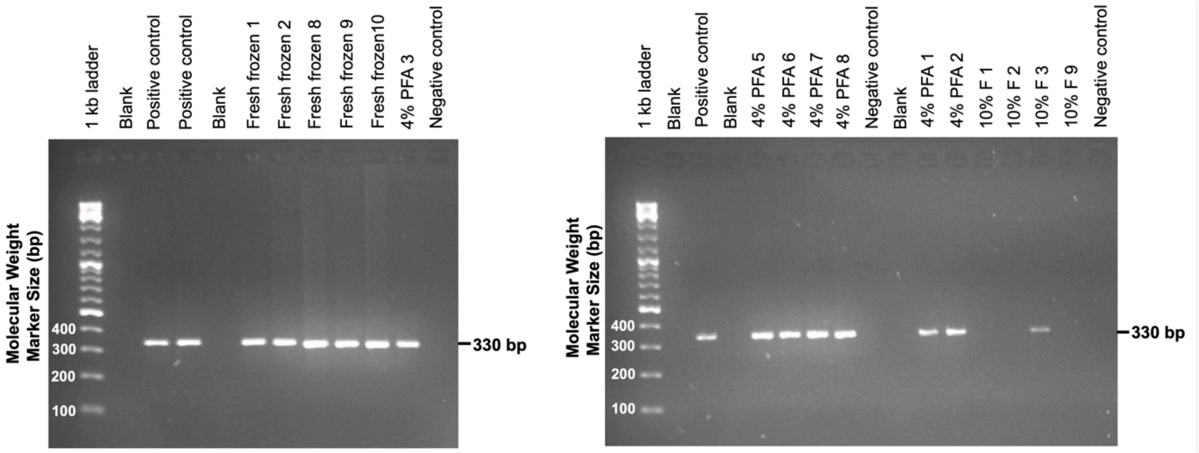

Figure S9 | Agarose gel electrophoresis results (2% w/v) showing PCR amplicons for exon 28 of mouse myosin binding protein C3 (Mybpc3) from fresh frozen, 4% PFA-fixed (4% PFA) and 10% formaldehyde-fixed (10% F) samples after cryo-X-PCI.

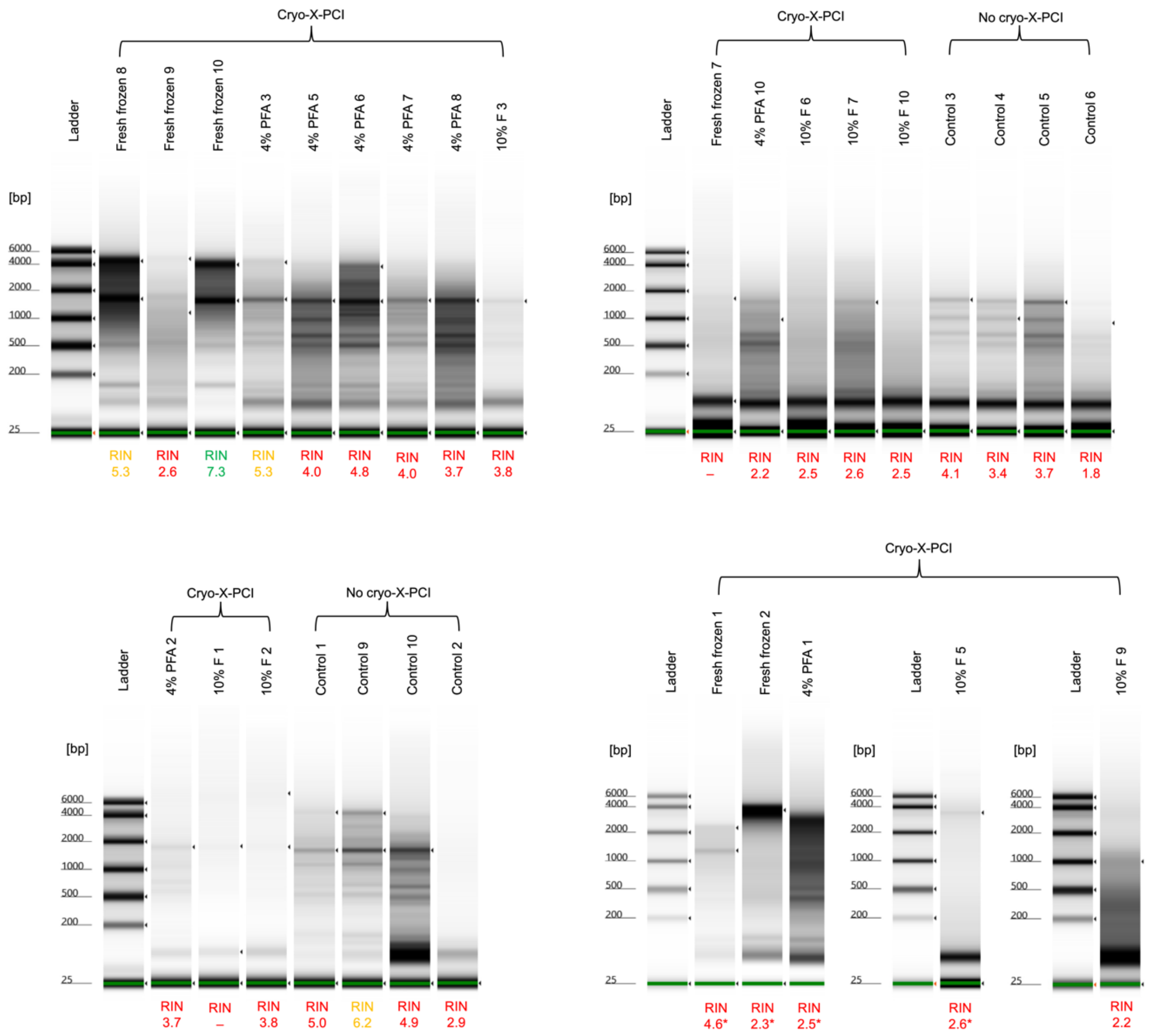

Figure S10 | Quality control TapeStation gels with RNA Integrity Number (RIN) values of RNA extracted from fresh frozen, 4% PFA-fixed (4% PFA), 10% formaldehyde-fixed (10% F) after cryo-X-PCI, and control mouse myocardial biopsies. Good RIN values are shown in green. Medium RIN values are shown in yellow. Poor RIN values are shown as red including those that could not be calculated due to very low concentrations of RNA. The asterisk (*) symbol represents samples that were run on High Sensitivity RNA Tape since they had very low RNA concentrations.

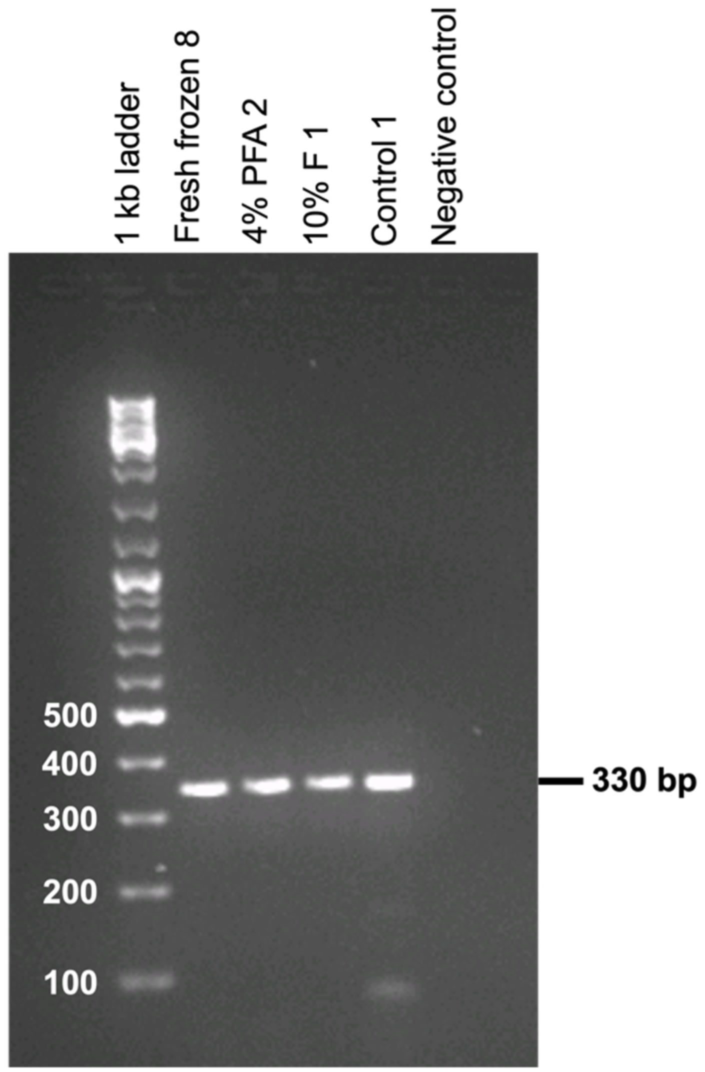

Figure S11 | Reverse transcription PCR results (2% w/v agarose gel electrophoresis). 11 µl of representative extracted RNA samples from mouse myocardial biopsies following cryo-X-PCI which were reverse transcribed using SuperScript™ IV First-Strand cDNA Synthesis Reaction kit (Invitrogen).

Supplementary Tables

Table S1 | Acquisition parameters used for synchrotron-based cryo-X-PCI of myocardial biopsies at TOMCAT X02DA beamline (Swiss Light Source).

Parameter	Value
Storage ring current	400 mA
Beam energy	21.002 keV
Propagation distance	60 mm
Magnification	10x
Effective pixel size	0.65 μm
Field of view	1.7 x 1.4 mm^2
Number of projections	1000
Number of darks	30
Number of flats	50
Exposure time	50 ms
Camera	PCO.Edge 5.5 sCMOS
Scintillator	LuAG:Ce 20 μm
Temperature range	-80°C
Cryojet sample flow	17.0 l/m
Cryojet shield flow	5.7 l/m
Scan time	3 minutes

Table S2 | Haematoxylin and eosin (H&E) frozen staining protocol for cryo-sections.

Step	Solution	Time	Mix
1	Start station	---	---
2	Tap water	00:10	+
3	Distilled water	01:00	+
4	Haematoxylin	00:45	+
5	Tap water	02:00	+
6	HCl/E-Alc	00:06	+
7	Tap water	03:00	+
8	Eosin	01:00	+
9	Tap water	00:10	---
10	E-Alc:70%	00:30	+
11	E-Alc:100%	01:00	+
12	E-Alc:100%	02:00	+
13	Xylene	02:00	+
14	Xylene	02:00	+
15	End station	---	---

Table S3 | Quality assessment and quantitation of DNA and RNA extracted from 22 wild-type mouse myocardial biopsies that underwent cryo-X-PCI, and 8 fresh frozen biopsies which did not have cryo-X-PCI. Measurements from Nanodrop and TapeStation are shown. High DNA integrity number (DIN) and RNA integrity number (RIN) values indicate high molecular weight and intact DNA and RNA, respectively, et vice versa. The asterisk (*) symbol represents samples that were subjected to High Sensitivity RNA ScreenTape assay due to very low RNA concentrations. 'X' denotes DIN and RIN values that could not be calculated due to very low concentrations. 'Fresh frozen' denotes hearts collected in RNAlater and snap frozen in liquid nitrogen. '4% PFA' denotes hearts collected in RNAlater then fixed in 4% paraformaldehyde (PFA) for 1h. '10% formaldehyde' denotes hearts collected in RNAlater then fixed in 10% formaldehyde (10% F) for 30 min. Control hearts, i.e., those that did not have cryo-X-PCI, were collected in RNAlater then snap frozen in liquid nitrogen.

Sample ID		Nanodrop measurements					TapeStation - Agilent 2200 TapeStation System			
		DNA concentration (ng/μl)	260/280	RNA concentration (ng/ul)	260/280	260/230	DNA concentration (ng/ul)	DIN	RNA concentration (ng/μl)	RIN
Cryo-X-PCI	Fresh frozen 1	20.1	2.00	2.6	1.24	0.06	9.16	6.6	2.95	4.6*
	Fresh frozen 2	37.8	2.23	3.2	0.97	0.45	21.6	7.5	16.8	2.3*
	Fresh frozen 3	Cryo-sectioned								
	Fresh frozen 4	Cryo-sectioned								
	Fresh frozen 5	Cryo-sectioned								
	Fresh frozen 6	Cryo-sectioned								
	Fresh frozen 7	26.5	1.88	7.5	1.76	0.187	22.7	6.6	8.23	X
	Fresh frozen 8	116.6	1.83	123	2.00	3.074	74.2	6.9	137.0	5.3
	Fresh frozen 9	41.1	1.85	35.2	2.05	0.88	21.9	7.2	36.5	2.6
	Fresh frozen 10	94.9	1.80	65.8	2.04	1.645	66.2	7.4	89.5	7.3
	4% PFA 1	1.0	0.74	19.9	1.70	0.36	2.84	X	28.4	2.5*
	4% PFA 2	1.5	7.38	106.8	1.17	0.83	12.3	6.4	14.8	3.7

	4% PFA 3	21.0	1.2	40.5	2.00	1.012	5.96	7.9	39.5	5.3
	4% PFA 4	Cryo-sectioned								
	4% PFA 5	29.8	1.57	84.3	1.99	2.107	12.7	6.8	83.9	4.0
	4% PFA 6	26.2	1.73	98.5	1.99	2.463	14.6	7.6	99.2	4.8
	4% PFA 7	30.2	1.65	42.3	1.98	1.059	16.9	7.2	41.3	4.0
	4% PFA 8	49.1	1.73	90.4	1.98	2.259	32.9	7.0	94.7	3.7
	4% PFA 9	Cryo-sectioned								
	4% PFA 10	23.9	1.56	81.8	1.94	2.046	44.1	5.3	61.7	2.2
	10% F 1	8.20	1.08	173.8	1.12	1.50	4.19	4.7	8.83	N/A
	10% F 2	2.60	1.12	147.8	1.22	0.81	3.04	2.2	10.9	3.8
	10% F 3	9.60	1.03	27.9	1.85	0.697	2.68	X	17.3	3.8
	10% F 4	Cryo-sectioned								
	10% F 5	3.70	1.16	2.3	1.48	0.057	1.19	X	0.696	2.6*
	10% F 6	3.40	1.01	16.9	1.51	0.422	0.940	X	21.1	2.5
	10% F 7	5.80	1.24	48.7	1.72	1.218	4.13	1.7	33.0	2.6
	10% F 8	Cryo-sectioned								
	10% F 9	17.4	0.92	20.4	1.77	0.51	2.30	X	4.98	2.2*
	10% F 10	4.30	1.00	55.8	1.47	1.395	1.98	X	20.7	2.5
**No cryo-X-PCI**	Control 1	31.1	1.65	168.4	2.03	1.40	19.4	7.5	121.0	5.0
	Control 2	13.3	2.38	42.9	0.87	0.21	9.3	7.0	11.0	2.9
	Control 3	27.8	1.81	40.5	1.80	1.012	12.3	7.7	28.1	4.1
	Control 4	23.1	1.64	69.8	2.03	1.744	17.6	7.6	54.6	3.4
	Control 5	18.3	1.86	75.7	1.88	1.892	20.0	7.2	48.1	3.7
	Control 6	43.4	1.77	19.6	1.70	0.491	22.3	6.7	10.7	1.8
	Control 7	Cryo-sectioned								
	Control 8	Cryo-sectioned								
	Control 9	14.3	1.94	107.7	1.21	0.33	10.5	7.0	27.2	6.2
	Control 10	12.6	2.11	43.8	1.85	0.97	8.63	6.8	44.1	4.9
